# A standardized framework for dietary intake data: implementation and monitoring of the 24-hour dietary recalls in the PROVEN-DIA trial

**DOI:** 10.3389/fnut.2026.1775650

**Published:** 2026-03-26

**Authors:** Ana Laura Fogaça, Thatiane Lopes Valentim Di Paschoale Ostolin, Raira Pagano, Angelica Barbosa Neres Santana, Danielle Cristina Fonseca, Lívia Tavares De Oliveira, Bárbara Shibuya Alves, Ângela Cristine Bersch-Ferreira

**Affiliations:** BP–A Beneficência Portuguesa de São Paulo–PROADI-SUS, São Paulo, Brazil

**Keywords:** clinical trials, data accuracy, data management, diet records, research design

## 1 Introduction

The primary approach for the prevention and management for the treatment of T2DM is lifestyle modification, which involves weight reduction and regular physical activity (1). In this context, accurate and standardized assessment of dietary intake is a critical component for evaluating lifestyle-based interventions. Repeated 24-h dietary recalls (24HR) are highly valuable due to their sensitivity to dietary change and their ability to support detailed analyses of nutrient intake, dietary patterns, and food processing levels (2).

However, the implementation of 24HR in multicenter trials presents important methodological challenges, particularly in settings characterized by heterogeneous populations and variability in professional training. These challenges are further amplified in studies conducted in low- and middle-income countries (LMICs), where cultural, socioeconomic, and dietary diversity can compromise data consistency and comparability if not adequately addressed.

The PROVEN-DIA trial exemplifies this complexity (3). Conducted in Brazil, a country marked by regional, cultural, socioeconomic, and dietary diversity, PROVEN-DIA required robust and scalable strategies to ensure the consistency, comparability, and quality of dietary intake data collected across multiple centers (3). Evidence from LMICs settings highlights the need for rigorous standardization of interviewer training, data collection procedures and quality control processes to adequately capture dietary intake amid cultural diversity and regional variability in food consumption patterns (4, 5). Accordingly, adopting standardized protocols is necessary to reduce measurement error and ensure data reliability (6).

To address these challenges, the PROVEN-DIA trial implemented a comprehensive framework that integrates standardized operating procedures, structured training, and continuous data quality monitoring across all participating sites. This framework supports consistent application of the 24HR method and enables robust analyses of nutrient intake, food group consumption, identification of ultra-processed and organic foods, diet quality indicators, and estimation of usual dietary intake.

In this article, we describe the standardized procedures and quality assurance mechanisms adopted for the 24HR data collection and entry in the PROVEN-DIA trial.

## 2 Methods

The PROVEN-DIA trial (ClinicalTrials.gov: NCT06426277) is a multicenter, parallel-group, randomized controlled clinical trial designed to evaluate the effectiveness of a structured lifestyle modification program in preventing type 2 diabetes mellitus (T2DM) among adults with prediabetes. The trial is conducted at 30 sites across Brazil's five macro-regions and aims to enroll 1,590 participants. The trial comprises three groups: Usual Treatment group, PROVEN-DIA (hybrid) and TelePROVEN-DIA (virtual). Recruitment began in Nov 2024, with completion expected in June 2026 and follow-up continuing until 2029. The PROVEN-DIA trial includes five scheduled assessment visits over the 3-year follow-up period, conducted at baseline and at 6, 12, 24 and 36 months. Trained staff collect clinical, anthropometric, biochemical, and behavioral data using standardized procedures across all study sites.

In this article, we focus specifically on the collection and quality assurance procedures for 24HR. Dietary intake is assessed at each study visit using two 24HR: the first is administered in person during the site visit with the support of a photographic manual, and the second is conducted by telephone on a non-consecutive day, within a maximum interval of seven days (7). In total, participants will complete 10 dietary recalls over the course of the trial. It is important to note that researchers across the 30 participating sites have diverse professional backgrounds, including dietitians, nurses, physical education, physicians, pharmacists, among others.

### 2.1 Data collection and electronic data entry

#### 2.1.1 Data collection

In the PROVEN-DIA Trial, dietary intake was assessed using a 24-h recall methodology patterned after the five-step USDA Automated Multiple-Pass Method (AMPM), adapted to the Brazilian research context (2). This method comprises: (1) a quick list, in which participants report all foods and beverages consumed during the previous 24 h; (2) probing for commonly forgotten foods or beverages; (3) identification of eating occasions and timing; (4) a detailed description cycle, including brand, preparation method, recipe, quantity, degree of food processing, and organic status; and (5) a final probing step to capture any additional items. The Automated Multiple-Pass Method is designed to improve recall accuracy and reduce measurement error in dietary assessment (2). More details were provided below.

#### 2.1.2 Data recording

Ten 24-h dietary recalls (24HR) are collected throughout the trial: two at baseline, and two at each follow-up time point (6 months, 1 year, 2 years, and 3 years). At each assessment, the first recall is conducted in person during the study visit, and the second is obtained by telephone on a non-consecutive day within a protocol-defined interval. All recalls are recorded in the individual case report form (CRF), which serves as the source document, and entered into the Vivanda^®^ system within seven days of collection. The same dietary assessment procedures are applied consistently across all study groups.

Vivanda^®^ is a secure, centralized, web-based system for dietary data management, where all reported foods, beverages, and energy-contributing supplements are recorded alongside mealtimes, classification of foods as ultra-processed and/or organic, and an indication of whether the recall day was typical or atypical of the participant's usual intake. System access is role-based and password-protected, with individual and exclusive credentials assigned to authorized researchers and access restricted by randomization groups, ensuring data confidentiality, traceability, and compliance with data protection regulations. Centralized administrative oversight enables monitoring of system changes and data-related actions.

### 2.2 Materials supporting data collection and data entry

As part of this training process, all trial staff completed the Good Clinical Practice (GCP) online course, ensuring compliance with international standards for ethical conduct, participant safety, and data integrity. All participating sites receive standardized in-person training during Site Initiation Visit (SIV) delivery by the coordination center.

As part of the in-person training, a dedicated 40-minute training session covered the five standardized steps of 24HR administration, including a hands-on workshop in which researchers practiced administering a 24HR. The training focused specifically on standardization of interview techniques to ensure methodological consistency across study sites. Core components included: (1) structured application of the five-step protocol; (2) standardized probing techniques to minimize omission of foods and beverages; (3) neutral interviewing strategies to reduce interviewer bias; (4) portion-size estimation using household measures and gram equivalents; and (5) documentation and coding of mixed dishes and regional preparations. During the hands-on component, interviewers conducted supervised mock interviews and received real-time feedback from the coordination team.

To support the standardized implementation of the 24HR, all study sites received a comprehensive set reference materials. These materials included instructional videos emphasizing standardized interviewing techniques to minimize common sources of error in dietary recalls, including inaccuracies in portion-size estimation using household measures, interviewer-induced response bias, and incomplete reporting of food preparation details (e.g., presence of added sugar). A detailed Standard Operating Manual was also provided, containing a dedicated section on 24HR administration, standardized probing techniques, documentation procedures, and operational guidance for use of the Vivanda^®^ electronic data capture system.

Additionally, a Guideline for the Standardization of Recipes and Foods were distributed to all sites, providing harmonized information on commonly consumed foods, regional preparations, portion-size estimation (including household measures and gram equivalents), and predefined coding criteria. This guideline served as a reference tool to assist interviewers in resolving uncertainties during data collection and to promote comparability of dietary data across Brazil's diverse dietary contexts.

To further support standardized data entry and system use, additional instructional materials were provided covering platform access, participant registration workflows, data entry procedures, and internal standardization processes. These resources aimed to minimize inter-site variability and ensure completeness, accuracy, and consistency of dietary data throughout the trial.

#### 2.2.1 Harmonization and customization of food and recipe data

A centralized harmonization process was implemented to balance cultural representativeness and methodological standardization across sites. The process included: (i) a structured workflow for registering new foods, recipes, and household measures via a centralized spreadsheet; and (ii) analyst review prior to inclusion to ensure consistency in nomenclature, portion sizes, and preparation assumptions (8).

The Guideline for the Standardization of Recipes and Foods also defined reference utensils and volumes, and established default decision rules for incomplete information (e.g., whole milk when unspecified; medium unit when fruit size was not reported). A photographic guide supported participant recall and interviewer portion estimation.

Given the sensitivity of sodium and fat estimates to cooking practices, harmonization of salt and oil content was applied through two standardized recipe profiles for staple dishes: lower-salt/oil (e.g., 1% salt and 2% soybean oil) and higher-salt/oil (e.g., 2% salt and 5% soybean oil). During 24HR interviews, adherence to recommended preparation practices was verified. When adherence was confirmed, the lower-salt/oil profile was selected, otherwise the higher-salt/oil profile was applied by default. This decision rule was consistently used for commonly consumed foods (e.g., rice, beans, meats, and vegetables).

These harmonization procedures were systematically incorporated into the verification-based and record-based monitoring processes and informed the study's conformity and error-rate indicators, particularly by standardizing assumptions related to food preparation practices.

### 2.3 Post-collection data quality and integrity monitoring

Data monitoring in the PROVEN-DIA trial is implemented as a continuous, centralized, and collaborative process involving the participating sites and the coordinating center's data management team. Dietary data are stored in a .txt format and exported in full or by study site and period using a standardized, semicolon-delimited structure, enabling automated monitoring routines and subsequent analyses of nutrient intake, food groups, dietary patterns and degree of food processing.

This framework comprises two complementary monitoring components. The first, verification-based monitoring, assessed consistency between source documents and data entered the Vivanda^®^ system. The second, record-based monitoring, relied exclusively on Vivanda^®^ data and predefined indicators of completeness and internal consistency to identify potential discrepancies or irregular patterns.

As part of the centralized monitoring strategy, a standardized Data Quality Report is generated periodically to consolidate predefined indicators of data completeness, internal consistency, and protocol adherence. This report serves as the primary operational tool for identifying discrepancies, guiding corrective actions, and providing structured feedback to participating sites.

Data quality was assessed using three central indicators reflecting correctness of data entry, consistency, and protocol adherence: (1) conformity rate (CR), represents the proportion of correctly entered food items relative to the total number of recorded items; (2) error rate (ER), the percentage of errors identified in the Data Quality Report; and (3) completeness rate (CoR), the proportion of participants with 24HR collected within the protocol-defined time window (9).

#### 2.3.1 Conformity monitoring

This component focuses on assessing conformity between data recorded in the CRF and the information transcribed into the Vivanda^®^ system. Coordinating centers periodically review a sample of records to ensure transcription accuracy and to identify potential deviations or missing information. Identified discrepancies are documented, and feedback is provided to the respective sites to guide data correction and reinforce adherence to the standardized data collection protocol.

Identified inconsistencies are classified according to their origin, enabling transparent documentation of data quality issues. Two main categories were defined: source document–related inconsistencies and system-related inconsistencies. Source document–related inconsistencies include lack of recipe standardization (e.g., failure to specify whether foods were home-prepared or commercially obtained, or whether standardized amounts of salt and oil were used), insufficient preparation details (e.g., cooking method, use of seasoning, or added sugar), and missing complementary information (e.g., classification as ultra-processed or organic foods). System-related inconsistencies include incorrect selection of foods or household measures, quantity errors, missing records, or discrepancies between the electronic database and the CRF (Supplementary Data 1).

For each site, an initial CR is calculated based on these indicators and recalculated after documented corrections, providing a transparent and auditable metric of data quality improvement over time. The CR is derived from the number of identified errors and is defined as the ratio between the total number of errors arising from both source document–related inconsistencies and system-related inconsistencies and the total number of recorded food items. It is expressed as a percentage ranging from 0 to 100% conformity. This indicator reflects the accuracy of data entry and the level of concordance between CRFs and electronic records. Identified discrepancies are documented and discussed during scheduled monitoring meetings. The indicator is recalculated six months after the initiation of follow-up of the first participant through a subsequent verification-based monitoring process, allowing assessment of data quality improvement over time and supporting the reproducibility of the monitoring process (Supplementary Data 2).


$$\begin{array}{l} CR\;\left(\%\right)=\left(1-\frac{Number\;of\;errors}{Total\;number\;of\;recorded\;food\;items}\right)\;x\;100 \end{array}$$

#### 2.3.2 Error monitoring

The second monitoring component relies exclusively on the electronic database and applies predefined and reproducible indicators of data completeness and internal consistency. A complete export of the electronic database is performed monthly to support automated verification. These routines are executed using a purpose-built script developed in RStudio (R) to systematically identify potential inconsistencies in the dataset. Automated and standardized routines perform systematic checks for missing values, logical inconsistencies between variables, and outliers in dietary intake data, generating periodic Data Quality Reports. These reports are independently reviewed by the central data management team and shared with participating sites, enabling transparent communication and traceable correction procedures. Based on these analyses, a monthly Data Quality Report is generated, consolidating all issues identified during the evaluation period.

The report includes a list of participants with delayed 24HR and identified inconsistencies. In addition, records with implausible total energy intake values (e.g., < 1,000 kcal or >3,000 kcal) are flagged for further review. In such cases, site interviewers are asked to verify the entries against the original paper CRF to identify potential data entry errors, particularly those related to portion-size transcription (e.g., extra digits in household measures or volumes). When transcription errors are confirmed, corrections are implemented following documentation to ensure traceability of data modifications. The report is made available through a dedicated SharePoint^®^ platform for each site, with exclusive access restricted to the respective site. Participants are identified exclusively by unique study IDs to ensure blinding. Research teams are notified via institutional email upon report release and are provided with a predefined deadline to correct pending issues or submit documented justifications, ensuring transparent and traceable data correction procedures.

Following the issuance of the Data Quality Report, the second quality indicator, the ER, is automatically generated within the R script (Supplementary Code 1). The ER is defined as the proportion of identified errors relative to the total number of food records for the evaluated period and is calculated as:


$$\begin{array}{l} ER\;\left(\%\right)=\;\left(\frac{Number\;of\;errors}{Total\;number\;of\;food\;records}\right)\;x\;100 \end{array}$$

This indicator quantifies the number of inconsistencies detected during data verification and serves as a standardized measure of data reliability and precision. Sites are responsible for correcting the identified discrepancies within predefined timelines after receiving the Data Quality Report.

#### 2.3.3 Completeness monitoring

Weekly monitoring is conducted to track each site's progress and to identify 24HR that should be available in the system but have not yet been entered. A standardized monitoring spreadsheet is generated and shared with coordinating center monitors, who are responsible for maintaining structured communication with participating sites and providing case-specific support when needed.

Based on the monitoring of delayed 24HR records, the third quality indicator, the CoR, is calculated weekly. The CoR represents the proportion of participants with 24HR collected within the protocol-defined time window and is calculated as:


$$\begin{array}{l} CoR\;\left(\%\right)=\;\left(\frac{Number\;of\;24HR\;recalls\;entered}{Total\;number\;of\;expected\;24HR\;recalls}\right)\;x\;100 \end{array}$$

This indicator reflects the completeness of dietary data entry for each site.

#### 2.3.4 Quality of data

**Figure 1:**
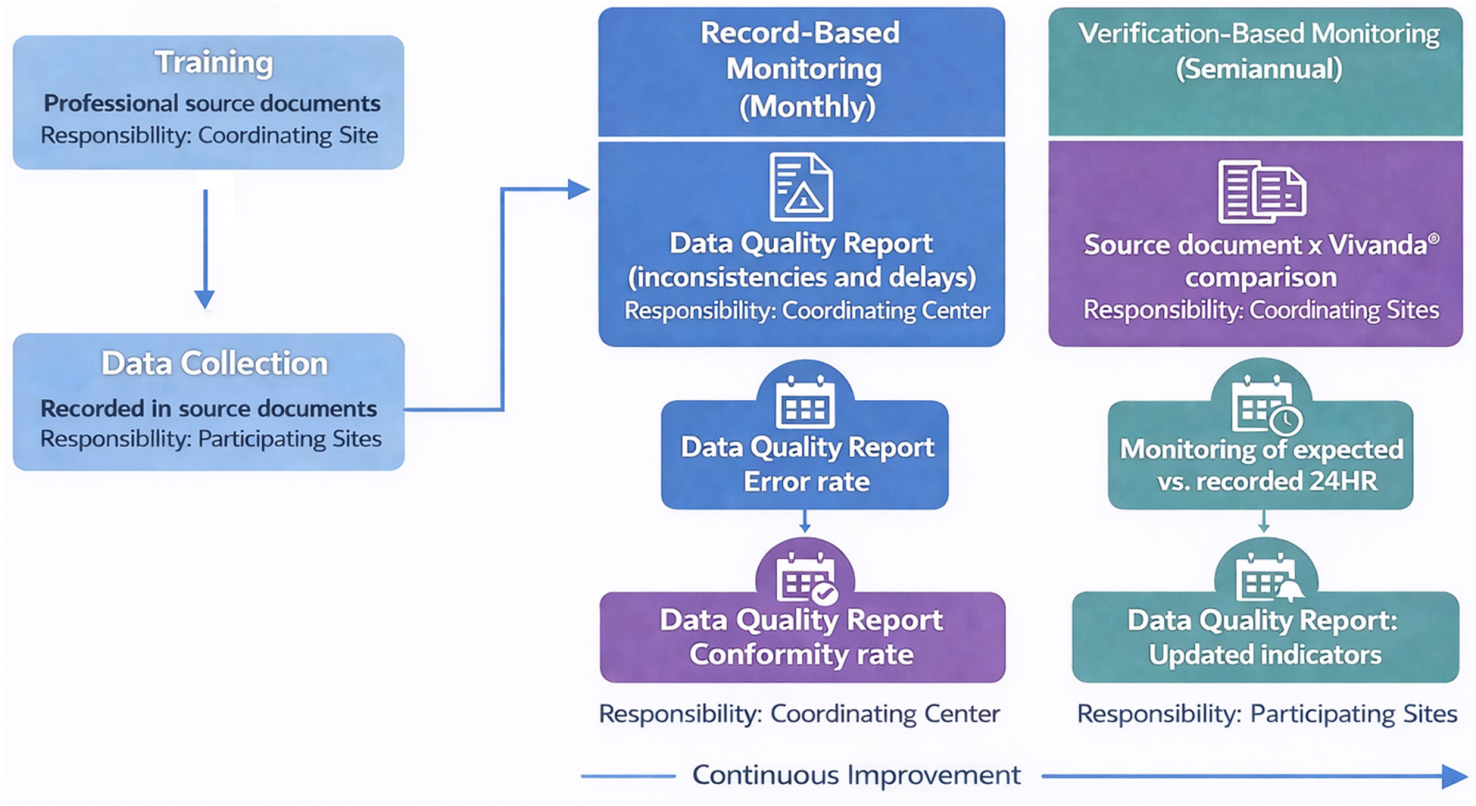
Flowchart of the data monitoring and quality reporting process for the PROVEN-DIA trial.

All the rates are continuously updated, enabling longitudinal monitoring of site performance and early detection of systematic error patterns (Figure 1). Record-based monitoring combines automated verification, manual review, and continuous communication between participating sites and the coordinating center.

A site with good data quality is expected to present an ER below 5% and both CoR and CR above 95%. All indicators are designed to support participating sites throughout the trial. When reductions in data quality are detected, site monitors receive targeted feedback and technical support through educational actions and follow-up meetings. Data quality assessment is conducted systematically and periodically. These indicators are recalculated at each monitoring cycle, providing an individual and comparative overview of site performance. This process supports the identification of persistent error trends, informs corrective actions, and contributes to standardization of 24HR collection procedures.

Indicators are calculated according to the monitoring schedule of each metric: CR during initial and follow-up monitoring, ER monthly through Data Quality Reports, and CoR weekly to track pending or delayed 24HR entries. To date, 122 reports have been issued, and approximately 1,562 are expected by 2029. Data completeness is reviewed weekly to monitor sites regarding records that have not yet been entered but are already within the expected timeframe. Each site is expected to achieve a CoR above 95% by the end of the trial, as the number of participants without 24HR data should be minimal after completion of follow-up.

Standardization of dietary data collection across multiple sites presents considerable methodological challenges, particularly in culturally and geographically diverse settings. While standardized training and quality control procedures for dietary data collection in multicenter trials have been previously described, few studies have reported comprehensive, ongoing monitoring frameworks with quantitative performance indicators, particularly in low- and middle-income country (LMIC) settings.

Previous multicenter nutrition studies have addressed these challenges through various approaches. The European Prospective Investigation into Cancer and Nutrition (EPIC) developed the standardized EPIC-Soft 24-h recall method adapted for pan-European dietary monitoring, incorporating built-in quality control procedures and structured interviewer training (10). This methodology was subsequently adapted for Latin American populations through the GloboDiet system, demonstrating feasibility of standardized approaches in diverse cultural contexts (11). The Women's Intervention Nutrition Study (WINS) established a quality assurance system including centralized review and feedback to sites, though details on specific metrics and correction procedures were limited (12). Similarly, the INTERMAP study described built-in quality checks and structured training across four countries, emphasizing the importance of systematic approaches to reduce measurement error (13). The Girls Health Enrichment Multisite Studies (GEMS) evaluated the impact of different review phases (local, coordinating center) on dietary recall data quality, finding that quality control procedures primarily reduced nutrient variances rather than shifting means (14). However, these studies focused predominantly on interviewer training and periodic review rather than continuous, metric-driven monitoring.

Harmonization of recipe and food composition data has received less systematic attention in the literature. Most studies describe the use of standardized food composition databases but provide limited detail on procedures for handling regional variations in food preparation or recipes. More recently, attention has shifted to automated quality checks and digital data capture systems. Guan et al. (15) applied source data verification to evaluate dietary intake coding quality and identified substantial discrepancy rates between diet history interviews and software outputs, highlighting the need for enhanced support tools and quality procedures. The ASCOT trial documented the need for additional data processing procedures to assess adherence to dietary guidelines, including manual adjustments to portion sizes and food item selection to improve accuracy (16). Similarly, the Nova24h web-based tool implemented standardized procedures to impute missing information about food processing level and preparation methods, using distribution patterns observed in the larger cohort (17). In low- and middle-income settings, Gibson et al. (6) emphasized that standardized protocols and quality control procedures are essential to minimize random errors in 24-h recall protocols, though they noted that implementation of such standardization remains rare across many regions.

The PROVEN-DIA trial addresses these challenges and extends previous approaches by implementing a comprehensive framework that combines several elements. First, a centralized harmonization process balances cultural representativeness with methodological standardization, including pre-defined decision rules for incomplete information (e.g., default milk types, fruit sizes) and dual recipe profiles for staple foods with varying salt and oil content, based on verification of adherence to recommended preparation practices. Second, a dual monitoring strategy combines verification-based review (comparing source documents to electronic data) with record-based monitoring (automated checks for completeness and consistency), supported by quantitative performance indicators (conformity rate, error rate, completeness rate) calculated continuously throughout the trial. This enables early detection of systematic errors and targeted corrective actions throughout the trial period. To our knowledge, this represents one of the first multicenter dietary trials in a Latin American LMIC setting to report such a comprehensive, metric-driven quality monitoring framework with predefined performance thresholds and iterative feedback mechanisms, contributing practical methodological guidance for future nutrition studies in complex, multicenter research environments.

This framework presents several strengths relevant to multicenter dietary trials. The dual monitoring approach (verification-based and record-based) enables early error detection, while quantitative performance indicators provide objective metrics for site evaluation. Centralized harmonization through predefined decision rules and dual recipe profiles addresses standardization challenges in culturally diverse settings. The web-based system with iterative feedback via Data Quality Reports ensures traceability, real-time monitoring, and continuous improvement. The framework proved scalable across 30 sites with varying research experience. However, important limitations were identified. The monitoring system is resource-intensive, requiring substantial staff time for manual verification and monthly report generation, a challenge for trials with limited budgets. Despite comprehensive training, sites may exhibit variable protocol adherence during early phases, highlighting the need for continuous support and the difficulty of maintaining consistency across diverse institutional cultures. Harmonization strategies, while reducing variability, may not fully capture regional food preparation diversity; default decision rules facilitate consistency but introduce assumptions that may not reflect all populations. Additionally, fundamental 24HR limitations, recall bias, social desirability, and underreporting persist regardless of quality control. The telephone-based second recall may introduce variability in portion size estimation compared to in-person interviews. Future trials may benefit from automated validation checks, artificial intelligence for anomaly detection, and periodic inter-site reliability assessments. Despite these limitations, this framework offers a systematic, replicable approach to enhancing dietary data quality in complex multicenter settings.

## 3 Conclusion

This article aimed to describe the framework, procedures, and monitoring strategies implemented to support the collection and entry 24HR data in the multicenter PROVEN-DIA trial. Rather than proposing a definitive solution to the well-recognized limitations of dietary assessment, this work documents the practical efforts undertaken to minimize systematic errors and improve transparency, consistency, and traceability of dietary data in a complex research setting.

Dietary intake assessment is inherently prone to measurement error, recall bias, and variability in reporting, particularly in large-scale, culturally diverse, and multicenter studies. Challenges such as heterogeneity in cooking practices, differences in interviewer experience, limitations of participant recall, and operational constraints remain unavoidable and were continuously encountered throughout the study. Accordingly, the strategies described herein were designed not to eliminate error, but to detect, document, and mitigate its impact through standardized training, harmonized decision rules, digital data capture, and continuous quality monitoring.

Within this context, the PROVEN-DIA trial experience demonstrates that a structured and adaptive framework (combining operational guidance, centralized oversight, and feedback loops) may enhance the reliability and comparability of 24HR data over time. Importantly, the monitoring routines and quality indicators are used iteratively to refine training and data collection practices, reinforcing a process of ongoing improvement rather than a fixed endpoint.

Although these strategies do not overcome all methodological limitations inherent to dietary assessment, they offer a scalable and replicable approach for implementing the 24HR method, including remote and telephone-based collection, in complex research environments. Beyond supporting internal data quality, this framework contributes practical methodological guidance for future nutrition studies and multicenter trials, highlighting the central role of operational planning, transparency, and continuous quality oversight in generating robust and reproducible dietary intake data.

## Data Availability

The original contributions presented in the study are included in the article/Supplementary material, further inquiries can be directed to the corresponding author.

## Supplementary material

Supplementary Data 1 Compliance monitoring framework table.

Supplementary Data 2 Compliance rate table.

Supplementary Code 1 R script for automating data quality reports.
