# Supplementary material for: A standardized framework for dietary intake data: implementation and monitoring of the 24-hour dietary recalls in the PROVEN-DIA trial
Source: Front Nutr. 2026 Mar 26;13:1775650. doi: 10.3389/fnut.2026.1775650 (PMC13061730; doi:10.3389/fnut.2026.1775650)
Supplement: Supplementary Code 1 — R script for automating data quality reports. [file Data_Sheet_1.pdf]

```
# ==============================================
# QUALITY REPORT – BY CENTER
# ==============================================

suppressPackageStartupMessages({
  library(tidyverse); library(readxl); library(openxlsx)
  library(lubridate); library(janitor); library(stringr); library(readr);
library(stringi)
})

# ----------------------------------------------
# Files / PATHS
# ----------------------------------------------
FILE_VIVANDA   <- "Vivandatotal.xlsx"
FILE_REDCAP    <-
"58214PROADIProgramaD-RandomizaoPBI_DATA_LABELS_2025-12-05_0733.csv"

downloads_path <- file.path(Sys.getenv("USERPROFILE"), "Downloads")
p_ <- function(x) file.path(downloads_path, x)
`%||%` <- function(a,b) if (!is.null(a) && length(a)>0 && !is.na(a)) a else b


norm_header <- function(x) {
  x <- as.character(x)
  x <- tolower(x)
  x <- stri_trans_general(x, "Latin-ASCII")
  x <- gsub("\\s+", "_", x)
  x <- gsub("[.]", "_", x)
  x <- trimws(x)
  x
}

# ----------------------------------------------
# NORMAL FUNCTIONS
# ----------------------------------------------
parse_date_robusto <- function(x){
  x <- as.character(x)
  suppressWarnings({
    y <- parse_date_time(
      x,
      orders = c("Ymd","Y-m-d","dmy","d/m/Y","mdy","m/d/Y","Ymd HMS","dmy
HMS","mdy HMS"),
      tz = "UTC")
  })
  as_date(y)
}
safe_as_date <- function(x){
  if (inherits(x, "Date")) return(x)
  if (is.numeric(x)) return(as.Date(x, origin = "1899-12-30"))
  parse_date_robusto(x)
}
id_normaliza <- function(id){
  id %>% as.character() %>% str_squish() %>% str_replace_all("\\s*-\\s*", "-")
}
```

```
id_valido <- function(id){
  id2 <- id_normaliza(id)
  str_detect(ifelse(is.na(id2), "", id2), "^\\d{4}-\\d{1,3}$")
}
inferir_id_contexto_vec <- function(ncentro, npac_digitado){
  id <- id_normaliza(npac_digitado)
  out <- rep(NA_character_, length(id))
  valid <- id_valido(id); out[valid] <- id[valid]
  s <- stringr::str_replace_all(id, "[^0-9]", "")
  mask1 <- !valid & !is.na(s) & nchar(s) >= 5
  centro1 <- ifelse(mask1, substr(s, 1, 4), NA_character_)
  num1    <- ifelse(mask1, substr(s, 5, nchar(s)), NA_character_)
  numi1   <- suppressWarnings(as.integer(num1))
  mask1b  <- mask1 & !is.na(numi1)
  out[mask1b] <- sprintf("%s-%d", centro1[mask1b], numi1[mask1b])
  remaining <- is.na(out)
  centroi <- suppressWarnings(as.integer(ncentro))
  numi2   <- suppressWarnings(as.integer(stringr::str_extract(id, "\\d{1,3}$")))
  mask2   <- remaining & !is.na(centroi) & !is.na(numi2)
  out[mask2] <- sprintf("%04d-%d", centroi[mask2], numi2[mask2])
  out
}
rotulo_r24h <- function(v,i) sprintf("%s - %s R24h", v, ifelse(i==1,"1º","2º"))
tvk <- function(v,i) paste0(v,"__",i)
ord_visita <- function(x){
  lvls <- c("V0 - 1º R24h","V0 - 2º R24h","V1 - 1º R24h","V1 - 2º R24h",
            "V2 - 1º R24h","V2 - 2º R24h","V3 - 1º R24h","V3 - 2º R24h",
            "V4 - 1º R24h","V4 - 2º R24h")
  factor(x, levels = lvls)
}
strip_accents <- function(x){
  y <- iconv(x, from = "", to = "ASCII//TRANSLIT")
  ifelse(is.na(y), x, y)
}
padroniza_grupo <- function(x){
  v <- str_to_lower(str_squish(as.character(x)))
  case_when(
    str_detect(v, "tele") ~ "TelePROVEN-DIA",
    str_detect(v, "proven") ~ "PROVEN-DIA",
    TRUE ~ "Grupo Controle"
  )
}

# ----------------------------------------------
# TIME
# ----------------------------------------------
centros_ref <- tribble(
  ~ncentro, ~nome_centro,
  1610,
"PUC_PR",1611,"HCPA",1612,"UFG",1613,"HUAC",1614,"HUCAM_UFES",1615,"HUPES_UFBA",

1616,"UnB",1617,"UNIFAP",1618,"IPES",1619,"LIGA",1621,"UFCSPA",1622,"UFMG",1623,
"UFMT",
```

```
1627,"UFRPE",1628,"UFSJ",1629,"UFT",1630,"UFV",1631,"UNIFOR",1632,"UNIVALI",1633
,"UPE"
)
visitas_tbl <- tibble(
  visita = c("V0","V1","V2","V3","V4"),
  offset_dias = c(0,195,365,735,1095)
)
ref_hoje <- Sys.Date()

# ---------------------------------------------
# REDCap
# ---------------------------------------------
stopifnot(file.exists(p_(ARQUIVO_REDCAP)))
redcap_raw <- suppressMessages(read_csv(p_(ARQUIVO_REDCAP), show_col_types =
FALSE)) %>% clean_names()
nm <- names(redcap_raw)
col_record_id <- nm[which(nm %in% c("record_id","record_id_","recordid"))][1]
col_dag       <- nm[which(str_detect(nm, "^data_access_group"))][1]
col_randata   <- nm[which(str_detect(nm, "data_da_randomiz"))][1]
col_grupo     <- nm[which(str_detect(nm, "^randomiza"))][1]
if (any(is.na(c(col_record_id,col_dag,col_randata,col_grupo))))
  stop("Colunas esperadas não encontradas no REDCap.")

redcap <- redcap_raw %>%
  transmute(
    npac_redcap = id_normaliza(.data[[col_record_id]]),
    dag         = as.character(.data[[col_dag]]),
    data_randomizacao = parse_date_robusto(.data[[col_randata]]),
    grupo_redcap      = as.character(.data[[col_grupo]])
  ) %>%
  mutate(
    ncentro = suppressWarnings(as.integer(str_sub(npac_redcap,1,4))),
    ncentro = if_else(is.na(ncentro), as.integer(9999), ncentro)
  ) %>% left_join(centros_ref, by="ncentro") %>%
  filter(!is.na(npac_redcap) & npac_redcap != "")

redcap_randomizados <- redcap %>% filter(!is.na(data_randomizacao)) %>%
distinct(npac_redcap, .keep_all = TRUE)
grupo_map <- redcap_randomizados %>% select(npac_redcap, grupo_redcap) %>%
distinct(npac_redcap, .keep_all = TRUE)
redcap_por_centro   <- redcap_randomizados %>% count(ncentro, name="n_redcap")

# ---------------------------------------------
# VIVANDA
# ---------------------------------------------
stopifnot(file.exists(p_(ARQUIVO_VIVANDA)))
viv_raw <- read_excel(p_(ARQUIVO_VIVANDA)) %>% clean_names()
viv <- viv_raw %>%
  mutate(
    ncentro        = suppressWarnings(as.integer(ncentro)),
    npac_digitado  = id_normaliza(npac),
    id_valido_flg  = id_valido(npac_digitado),
    data           = safe_as_date(data),
    qtdemedcaseira =
```

```
suppressWarnings(as.numeric(str_replace_all(as.character(qtdemedcaseira),
",",".")))),
    qtdegramas     = suppressWarnings(as.numeric(qtdegramas)),
    kcal           = suppressWarnings(as.numeric(kcal)),
    status24h      = as.character(status24h),
    visita         = as.character(visita),
    grupo_viv      = as.character(grupo)
  ) %>% left_join(centros_ref, by="ncentro")

# NORMALIZAÇÃO VIVANDA
viv_norm <- viv %>%
  mutate(
    visita_norm = case_when(
      str_detect(visita,"\\bV0\\b")~"V0", str_detect(visita,"\\bV1\\b")~"V1",
      str_detect(visita,"\\bV2\\b")~"V2", str_detect(visita,"\\bV3\\b")~"V3",
      str_detect(visita,"\\bV4\\b")~"V4", TRUE~NA_character_
    ),
    ord_txt = case_when(
      str_detect(visita, regex("\\b1\\s*(?:º|°|o)?\\s*(?:R?24h)?\\b", TRUE)) ~
1L,
      str_detect(visita, regex("\\b2\\s*(?:º|°|o)?\\s*(?:R?24h)?\\b", TRUE)) ~
2L,
      TRUE ~ NA_integer_
    )
  ) %>%
  group_by(npac_digitado, visita_norm) %>%
  arrange(data, .by_group = TRUE) %>%
  mutate(
    seq_ord   = if_else(!is.na(visita_norm), dense_rank(coalesce(data,
as.Date("1900-01-01"))), NA_integer_),
    ord_final = coalesce(ord_txt, if_else(seq_ord %in% c(1L,2L), seq_ord,
NA_integer_)),
    tipo_visita = if_else(!is.na(visita_norm) & !is.na(ord_final),
rotulo_r24h(visita_norm, ord_final), NA_character_),
    tv_key     = if_else(!is.na(visita_norm) & !is.na(ord_final),
tvk(visita_norm, ord_final), NA_character_)
  ) %>% ungroup()

viv_valid <- viv_norm %>%
  filter(id_valido_flg) %>%
  transmute(ncentro, nome_centro, npac = npac_digitado, visita_norm, ord =
ord_final,
            tipo_visita, tv_key, data, status24h, alimento, medcaseira,
            qtdemedcaseira, qtdegramas, kcal, grupo_viv)

digitados_inferidos_keys <- viv_norm %>%
  filter(!id_valido_flg, !is.na(npac_digitado), npac_digitado != "",
!is.na(tv_key)) %>%
  transmute(npac = inferir_id_contexto_vec(ncentro, npac_digitado), tv_key) %>%
  filter(!is.na(npac)) %>% distinct()

viv_kcal <- viv_valid %>%
  group_by(npac, tipo_visita, data) %>%
  summarise(kcal_total = sum(suppressWarnings(as.numeric(kcal)), na.rm=TRUE),
```

```
.groups="drop")

status_lookup <- viv_valid %>%
  mutate(stu = str_to_lower(str_trim(status24h))) %>%
  group_by(npac, tipo_visita, data) %>%
  summarise(status_rel = case_when(
    any(stu=="parcialmente") ~ "Parcialmente",
    any(stu=="finalizado")   ~ "Finalizado",
    TRUE ~ NA_character_
  ), .groups="drop")

# ---------------------------------------------
# QUERYS
# ---------------------------------------------
viv_qty_flags <- viv_valid %>%
  mutate(
    qtdegramas     = as.numeric(qtdegramas),
    qtdemedcaseira = as.numeric(str_replace_all(as.character(qtdemedcaseira),
",",".")),
    med_norm0 = as.character(medcaseira),
    med_norm  = med_norm0 %>% strip_accents() %>% tolower() %>%
str_replace_all("[()\\[\\].,;-]", " ") %>% str_squish(),
    is_ml = str_detect(med_norm,
"\\b(ml|m\\s*l|mili?litro(s)?|milli?liter(s)?)\\b"),
    is_g  = str_detect(med_norm, "\\b(g|gr|grama(s)?|gram(s)?)\\b"),
    flag_1g_mc_gt10_not_ml = !is.na(qtdegramas) & qtdegramas == 1 &
!is.na(qtdemedcaseira) & qtdemedcaseira > 10 & !is_ml,
    flag_mc_gt10_not_g_ml  = !is.na(qtdemedcaseira) & qtdemedcaseira > 10 &
!is_g & !is_ml,
    flag_qtd_erro = flag_1g_mc_gt10_not_ml | flag_mc_gt10_not_g_ml,
    item_label = if_else(flag_qtd_erro,
                         paste0(alimento,
                                ifelse(!is.na(qtdegramas), paste0(" — ",
qtdegramas, " g"), ""),
                                ifelse(!is.na(qtdemedcaseira),
                                       paste0(", ", as.integer(qtdemedcaseira),
" ", medcaseira), "")),
                         NA_character_)
  ) %>%
  filter(flag_qtd_erro, !is.na(tipo_visita), !is.na(data)) %>%
  select(npac, tipo_visita, tv_key, data, item_label)

err_qtd <- viv_qty_flags %>%
  group_by(npac, tipo_visita, tv_key, data) %>%
  summarise(descricao = paste(item_label, collapse = "; "), .groups = "drop")
%>%
  mutate(tipo_erro = "Quantidade")

key_map <- viv_valid %>% distinct(npac, tipo_visita, tv_key)

err_kcal <- viv_kcal %>%
  filter(!is.na(kcal_total), kcal_total <= 1000 | kcal_total >= 3000) %>%
  left_join(key_map, by=c("npac","tipo_visita")) %>%
  mutate(tipo_erro="Calorias",
```

```
        descricao = "R24h com kcal fora da faixa (≤1000 ou ≥3000)")

err_status <- viv_valid %>%
  mutate(status_up = str_to_upper(str_trim(status24h))) %>%
  filter(status_up == "Parcialmente", !is.na(tipo_visita), !is.na(data)) %>%
  distinct(npac, tipo_visita, tv_key, data) %>%
  mutate(tipo_erro="Status", descricao="R24h não finalizado (PARCIALMENTE)")

err_participante <- viv_norm %>%
  filter(!id_valido_flg, !is.na(npac_digitado), npac_digitado != "",
!is.na(tv_key)) %>%
  transmute(npac = npac_digitado, tipo_visita, tv_key, data = as.Date(NA),
            ncentro_part = ncentro, nome_centro_part = nome_centro,
            tipo_erro = "Participante",
            descricao = "Formato de ID inválido — corrigir no Vivanda") %>%
  distinct(npac, tipo_visita, tv_key, .keep_all = TRUE)

# LATES
visitas_cross <- visitas_tbl %>% tidyr::crossing(i = 1:2)
agenda_esperada <- redcap_randomizados %>%
  tidyr::crossing(visitas_cross) %>%
  mutate(
    data_base = data_randomizacao + days(offset_dias),
    anchor1 = if_else(visita == "V0", data_randomizacao + days(7), data_base),
    janela_inicio = case_when(
      i == 1 & visita == "V0" ~ data_randomizacao + days(1),
      i == 1 & visita != "V0" ~ anchor1,
      i == 2                  ~ anchor1 + days(1)
    ),
    janela_fim = case_when(
      i == 1 & visita == "V0" ~ data_randomizacao + days(7),
      i == 1 & visita != "V0" ~ anchor1,
      i == 2                  ~ anchor1 + days(7)
    ),
    tipo_visita   = rotulo_r24h(visita, i),
    tv_key        = tvk(visita, i),
    deve_existir  = Sys.Date() >= janela_inicio
  ) %>%
  select(npac_redcap, ncentro, nome_centro, grupo_redcap,
         visita, i, tipo_visita, tv_key,
         data_randomizacao, janela_inicio, janela_fim, deve_existir)

digitados_valid_keys <- viv_valid %>% filter(!is.na(tv_key)) %>% distinct(npac,
tv_key)

rec_base <- agenda_esperada %>%
  filter(deve_existir) %>%
  transmute(npac=npac_redcap, tipo_visita, tv_key, data=as.Date(NA),
            tipo_erro="Atrasado", descricao="R24h ausente / atrasado",
            ncentro, nome_centro, data_randomizacao, janela_inicio, janela_fim,
grupo_redcap) %>%
  anti_join(digitados_valid_keys,     by=c("npac","tv_key")) %>%
  anti_join(digitados_inferidos_keys, by=c("npac","tv_key")) %>%
  distinct(npac, tipo_visita, tv_key, data, tipo_erro, .keep_all = TRUE)
```

```
# ---------------------------------------------
# CONSOLIDATION > FINAL TABLE (incons_all)
# ---------------------------------------------
viv_grupo_lookup <- viv_valid %>%
  transmute(npac, grupo_viv = padroniza_grupo(grupo_viv)) %>%
  filter(!is.na(npac) & npac != "") %>%
  group_by(npac) %>% summarise(grupo_viv = dplyr::first(na.omit(grupo_viv)),
.groups = "drop")

id_infer_map <- viv_norm %>%
  filter(!id_valido_flg, !is.na(npac_digitado), npac_digitado != "") %>%
  transmute(npac = npac_digitado,
            npac_inferido = inferir_id_contexto_vec(ncentro, npac_digitado)) %>%
  left_join(redcap_randomizados %>% select(npac_redcap, grupo_redcap),
            by = c("npac_inferido" = "npac_redcap")) %>%
  transmute(npac, grupo_redcap_infer = grupo_redcap) %>%
  group_by(npac) %>% summarise(grupo_redcap_infer =
dplyr::first(na.omit(grupo_redcap_infer)), .groups = "drop")

incons_kq <- bind_rows(
  err_kcal   %>% select(npac, tipo_visita, tv_key, data, tipo_erro, descricao),
  err_qtd    %>% select(npac, tipo_visita, tv_key, data, tipo_erro, descricao),
  err_status %>% select(npac, tipo_visita, tv_key, data, tipo_erro, descricao)
) %>% filter(id_valido(npac))

incons_kq2 <- incons_kq %>%
  left_join(viv_kcal,       by=c("npac","tipo_visita","data")) %>%
  left_join(status_lookup,  by=c("npac","tipo_visita","data"))

incons_rec <- rec_base %>%
  transmute(npac, tipo_visita, tv_key, data, tipo_erro, descricao,
            kcal_total = 0, status_rel = "Atrasado",
            ncentro_rec = ncentro, nome_centro_rec = nome_centro)

incons_all_raw <- bind_rows(
  incons_kq2 %>% mutate(ncentro_rec = NA_integer_, nome_centro_rec =
NA_character_, ncentro_part = NA_integer_, nome_centro_part = NA_character_),
  err_participante %>% mutate(kcal_total = NA_real_, status_rel = NA_character_,
ncentro_rec = NA_integer_, nome_centro_rec = NA_character_),
  incons_rec
) %>%
  distinct(npac, tipo_visita, tv_key, data, tipo_erro, .keep_all = TRUE) %>%
  left_join(grupo_map,         by = c("npac" = "npac_redcap")) %>%
  left_join(viv_grupo_lookup, by = "npac") %>%
  left_join(id_infer_map,      by = "npac") %>%
  mutate(
    centro_from_id = suppressWarnings(as.integer(str_sub(npac,1,4))),
    Centro = coalesce(ncentro_part, ncentro_rec, centro_from_id),
    `Nome Centro` = coalesce(nome_centro_part, nome_centro_rec)
  ) %>%
  distinct(npac, tipo_visita, tv_key, data, tipo_erro, Centro, `Nome Centro`,
.keep_all = TRUE)
```

```
part_single_global <- incons_all_raw %>%
  filter(tipo_erro == "Participante") %>%
  arrange(npac, tipo_visita) %>%
  group_by(npac) %>% slice(1) %>% ungroup()

incons_all <- bind_rows(
  incons_all_raw %>% filter(tipo_erro != "Participante"),
  part_single_global
) %>%
  mutate(
    Grupo = padroniza_grupo(coalesce(grupo_redcap, grupo_viv,
grupo_redcap_infer,
                                     if_else(tipo_erro == "Atrasado",
"Atrasado", NA_character_))),
    `Status 24h` = status_rel,
    `Calorias`   = kcal_total
  ) %>%
  select(
    Centro, `Nome Centro`, ID = npac, Data = data,
    `Tipo Visita` = tipo_visita, Grupo, `Tipo Erro` = tipo_erro,
    `Status 24h`, `Calorias`, `Descrição Erro` = descricao
  ) %>%
  arrange(desc(id_valido(ID)), `Tipo Erro` == "Participante", Centro, ID, `Tipo
Visita`, `Tipo Erro`)

# ---------------------------------------------
# FUNCTIONS STILES
# ---------------------------------------------
col_primary <- "#0F766E"; col_light <- "#E6F4EA"
st_title <- createStyle(fontName="Calibri", fontSize=18, textDecoration="bold",
                        fgFill=col_light, fontColour=col_primary,
halign="center", valign="center")
st_sub   <- createStyle(fontName="Calibri", fontSize=12, fontColour=col_primary,
halign="center", valign="center")
st_header<- createStyle(fontName="Calibri", fontSize=11, textDecoration="bold",
                        fgFill=col_primary, fontColour="#FFFFFF",
halign="center")
st_border <- createStyle(border="TopBottomLeftRight", borderStyle="thin",
borderColour="#000000")
st_date  <- createStyle(numFmt="DD/MM/YYYY")
st_row   <- createStyle(fgFill=col_light)

write_title_block <- function(wb, sheet, title, sub1=NULL, sub2=NULL, sub3=NULL,
total_cols=12, start_row=1){
  addWorksheet(wb, sheet)
  writeData(wb, sheet, title, startRow=start_row, startCol=1)
  if(!is.null(sub1)) writeData(wb, sheet, sub1, startRow=start_row+1,
startCol=1)
  if(!is.null(sub2)) writeData(wb, sheet, sub2, startRow=start_row+2,
startCol=1)
  if(!is.null(sub3)) writeData(wb, sheet, sub3, startRow=start_row+3,
startCol=1)
  mergeCells(wb, sheet, cols=1:total_cols, rows=start_row)
  if(!is.null(sub1)) mergeCells(wb, sheet, cols=1:total_cols, rows=start_row+1)
```

```
  if(!is.null(sub2)) mergeCells(wb, sheet, cols=1:total_cols, rows=start_row+2)
  if(!is.null(sub3)) mergeCells(wb, sheet, cols=1:total_cols, rows=start_row+3)
  addStyle(wb, sheet, st_title, rows=start_row, cols=1, gridExpand=TRUE)
  if(!is.null(sub1)) addStyle(wb, sheet, st_sub, rows=start_row+1, cols=1,
gridExpand=TRUE)
  if(!is.null(sub2)) addStyle(wb, sheet, st_sub, rows=start_row+2, cols=1,
gridExpand=TRUE)
  if(!is.null(sub3)) addStyle(wb, sheet, st_sub, rows=start_row+3, cols=1,
gridExpand=TRUE)
}

style_table <- function(wb, sheet, df, startRow=5){
  addStyle(wb, sheet, st_header, rows=startRow, cols=1:ncol(df),
gridExpand=TRUE)
  addFilter(wb, sheet, rows=startRow, cols=1:ncol(df))
  freezePane(wb, sheet, firstActiveRow=startRow+1, firstActiveCol=1)
  n <- nrow(df)
  if(n>0){
    addStyle(wb, sheet, st_row, rows=(startRow+1):(startRow+n), cols=1:ncol(df),
gridExpand=TRUE, stack=TRUE)
    addStyle(wb, sheet, st_border, rows=(startRow+1):(startRow+n),
cols=1:ncol(df), gridExpand=TRUE, stack=TRUE)
  }
  date_cols <- which(str_detect(tolower(names(df)), "data"))
  if(length(date_cols)>0 && nrow(df)>0)
    addStyle(wb, sheet, st_date, rows=(startRow+1):(startRow+n), cols=date_cols,
gridExpand=TRUE, stack=TRUE)
  setColWidths(wb, sheet, cols=1, widths=10)
  if(ncol(df) > 1) setColWidths(wb, sheet, cols=2, widths=12)
  if(ncol(df) > 2) setColWidths(wb, sheet, cols=3:ncol(df), widths="auto")
  pageSetup(wb, sheet, orientation="landscape", fitToWidth=TRUE)
}

apply_dropdown_por_linha <- function(wb, sheet, df, startRow = 5){
  col_resp <- which(names(df) == "Resposta")
  if (length(col_resp) != 1 || nrow(df) == 0) return(invisible())
  tipos <- tolower(trimws(as.character(df$`Tipo Erro`)))
  for (i in seq_len(nrow(df))) {
    val <- switch(tipos[i],
                  "status"       = '"Status corrigido"',
                  "calorias"     = '"Kcal correta,R24h corrigido"',
                  "quantidade"   = '"Quantidade correta,Quantidade corrigida"',
                  "participante" = '"ID corrigido"',
                  "recordatório" = '"R24h incluído"',
                  "recordatorio" = '"R24h incluído"',
                  "atrasado"     = '"R24h incluído,R24h não coletado"',
                  NULL)
    if (!is.null(val)) dataValidation(wb, sheet, cols = col_resp, rows =
startRow + i,
                                      type = "list", value = val, allowBlank =
TRUE)
  }
}
```

```
colorize_rows_by_tipoerro <- function(wb, sheet, df, startRow = 5){
  palette <- c("#E3F7E8","#C9F0D3","#B0E9BF","#96E2AA","#7DDB96",
               "#63D482","#4ACE6D","#30C758","#27B84F","#1FA945",
               "#179A3C","#0F8B32")
  if (!("Tipo Erro" %in% names(df)) || nrow(df)==0) return(invisible())
  fixed <- c(
    "atrasado"     = "#B0E9BF",
    "status"       = "#C9F0D3",
    "calorias"     = "#7DDB96",
    "quantidade"   = "#B0E9BF",
    "participante" = "#30C758",
    "recordatório" = "#E3F7E8",
    "recordatorio" = "#E3F7E8"
  )
  tipos_raw <- tolower(trimws(df$`Tipo Erro`))
  unknown <- setdiff(unique(tipos_raw), names(fixed))
  if (length(unknown) > 0) {
    free_cols <- setdiff(palette, unname(fixed))
    extra_map <- stats::setNames(
      if (length(free_cols)==0) rep("#96E2AA", length(unknown))
      else rep_len(free_cols, length(unknown)),
      unknown
    )
    fixed <- c(fixed, extra_map)
  }
  fills <- fixed[tipos_raw]
  for (i in seq_len(nrow(df))) {
    addStyle(wb, sheet, style = createStyle(fgFill = fills[i] %||% "#E3F7E8"),
             rows = startRow + i, cols = 1:ncol(df), gridExpand = TRUE, stack =
TRUE)
  }
  invisible()
}

# ============================================
# # SITES | ERROR RATE
# ============================================


resumo_total_linhas <- viv_norm %>%
  mutate(Centro = suppressWarnings(as.integer(ncentro))) %>%
  filter(!is.na(Centro)) %>%
  count(Centro, name = "total_registros")


resumo_total_erros <- incons_all %>%
  mutate(te = tolower(trimws(`Tipo Erro`))) %>%
  filter(te %in% c("quantidade", "medida caseira", "status", "participante"))
%>%
  filter(!is.na(Centro)) %>%
  count(Centro, name = "total_erros")


df_taxas_final <- centros_ref %>%
```

```
  rename(Centro = ncentro) %>%
  left_join(resumo_total_linhas, by = "Centro") %>%
  left_join(resumo_total_erros, by = "Centro") %>%
  mutate(
    total_registros = coalesce(total_registros, 0L),
    total_erros     = coalesce(total_erros, 0L),

    perc = if_else(total_registros > 0,
                   round(100 * total_erros / total_registros, 1),
                   0)
  ) %>%

  transmute(
    CENTRO = nome_centro,
    `TAXA DE ERRO` = paste0(perc, "%")
  ) %>%
  arrange(CENTRO)


arquivo_taxas <- p_(paste0("Resumo_Taxas_Erro_", format(Sys.Date(), "%Y-%m-%d"),
".xlsx"))

write.xlsx(df_taxas_final, file = arquivo_taxas)

message(paste("Arquivo de taxas gerado com sucesso em:", arquivo_taxas))


# --------------------------------------------
# GENERATE REPORTS BY CENTER
# --------------------------------------------
viv_por_centro <- bind_rows(
  viv_valid %>% select(npac) %>% mutate(src="ok"),
  digitados_inferidos_keys %>% select(npac) %>% mutate(src="infer")
) %>%
  mutate(ncentro = suppressWarnings(as.integer(str_sub(npac,1,4)))) %>%
  semi_join(redcap_randomizados %>% distinct(npac_redcap), by = c("npac" =
"npac_redcap")) %>%
  distinct(ncentro, npac) %>% count(ncentro, name = "n_vivanda")

centros_list <- incons_all %>%
  filter(!is.na(Centro)) %>%
  group_by(Centro) %>%
  summarise(`Nome Centro` = {
    nc <- na.omit(`Nome Centro`); if (length(nc) > 0) nc[1] else NA_character_
  }, .groups = "drop") %>%
  left_join(centros_ref, by = c("Centro" = "ncentro")) %>%
  transmute(Centro, `Nome Centro` = coalesce(`Nome Centro`, nome_centro,
paste0("Centro_", Centro))) %>%
  arrange(Centro)

if (nrow(centros_list) == 0) {
  message("Não há inconformidades para gerar relatórios por centro.")
} else {
  for (i in seq_len(nrow(centros_list))) {
```

```
    ccode <- centros_list$Centro[i]
    cnome <- centros_list$`Nome Centro`[i] %||% paste0("Centro_", ccode)

    inc_c_full <- incons_all %>%
      filter((!is.na(Centro) & Centro == ccode) | stringr::str_sub(ID, 1, 4) ==
sprintf("%04d", ccode))
    if (nrow(inc_c_full) == 0) { message("Sem inconformidades para o centro: ",
cnome); next }

    part_single <- inc_c_full %>%
      filter(`Tipo Erro` == "Participante") %>%
      mutate(ordv = ord_visita(`Tipo Visita`)) %>%
      arrange(ID, ordv) %>%
      group_by(ID) %>% slice(1) %>% ungroup() %>% select(-ordv)

    inc_c_raw <- bind_rows(
      inc_c_full %>% filter(`Tipo Erro` != "Participante"),
      part_single
    ) %>% arrange(desc(id_valido(ID)), `Tipo Erro` == "Participante", ID, `Tipo
Visita`)

    inc_c <- inc_c_raw %>%
      select(ID, Data, `Tipo Visita`, Grupo, `Tipo Erro`, `Status 24h`,
`Calorias`, `Descrição Erro`) %>%
      mutate(
        Grupo = padroniza_grupo(Grupo),
        Resposta = NA_character_,
        `Data Resolução` = as.Date(NA),
        `Observações` = NA_character_,
        `Dúvidas e Sugestões` = NA_character_
      )


    total_linhas <- viv_norm %>%
      mutate(centro = suppressWarnings(as.integer(ncentro))) %>%
      filter(centro == ccode) %>%
      nrow()

    tipos_considerados <- c("quantidade","medida
caseira","status","participante")
    erros_considerados <- inc_c_raw %>%
      mutate(te = tolower(trimws(`Tipo Erro`))) %>%
      filter(te %in% tipos_considerados) %>%
      nrow()

    taxa_erro <- ifelse(total_linhas > 0, round(100 * erros_considerados /
total_linhas, 1), NA_real_)
    taxa_txt <- paste0("Taxa de erro (quantidade/medida caseira, status,
participante): ",
                       ifelse(is.na(taxa_erro), "N/D", paste0(taxa_erro, "%")),
                       " — erros: ", erros_considerados, " de ", total_linhas,
                       " registros no Vivanda.")

    n_viv_c <- viv_por_centro %>% filter(ncentro == ccode) %>% pull(n_vivanda);
```

```
    n_viv_c <- ifelse(length(n_viv_c)==0,0,n_viv_c)

    n_red_c <- redcap_por_centro %>% filter(ncentro == ccode) %>%
pull(n_redcap);
    n_red_c <- ifelse(length(n_red_c)==0,0,n_red_c)

    comparativo_centro <- paste0("COMPARATIVO ", cnome, " (Randomizados):
Vivanda (", n_viv_c, ") | REDCap (", n_red_c, ")")

    arq_centro <- p_(sprintf("%s_relatoriodequalidade_%s_%s.xlsx", cnome,
                             format(ref_hoje, "%b") %>% tolower(),
                             format(ref_hoje, "%Y")))

    # ---------------------------
    # FILTER LINES
    # ---------------------------

    controle_file <- p_("Controle_inconformidades_por_centro.xlsx")

    if(file.exists(controle_file)){


      aba_controle <- tryCatch({
        read_excel(controle_file, sheet = cnome) %>% clean_names()
      }, error = function(e) {
        message("Não foi possível ler a aba do centro ", cnome, " no arquivo de
controle.")
        return(NULL)
      })

      if(!is.null(aba_controle)){


        names(aba_controle) <- norm_header(names(aba_controle))


        if(all(c("id","tipo_erro","calorias","resposta") %in%
names(aba_controle))){


          aba_controle <- aba_controle %>% mutate(id = as.character(id))


          linhas_respondidas <- aba_controle %>%
            filter(!is.na(resposta) & resposta != "") %>%
            select(id, tipo_erro, calorias)


          linhas_respondidas <- linhas_respondidas %>%
            mutate(
              id = as.character(id),
              tipo_erro = as.character(tipo_erro),
              calorias = as.character(calorias)
            )
```

```
          inc_c <- inc_c %>%
            mutate(
              ID = as.character(ID),
              `Tipo Erro` = as.character(`Tipo Erro`),
              Calorias = as.character(Calorias)
            )


          inc_c <- inc_c %>%
            anti_join(linhas_respondidas, by = c("ID" = "id",
                                                 "Tipo Erro" = "tipo_erro",
                                                 "Calorias" = "calorias"))


          message(sprintf("   → Centro %s: %d linhas removidas por já terem
resposta", cnome,
                          nrow(inc_c_raw) - nrow(inc_c)))
        } else {
          message("Colunas obrigatórias não encontradas na aba de controle do
centro ", cnome)
        }
      }
    } else {
      message("Arquivo de controle não encontrado em: ", controle_file)
    }


    wb2 <- createWorkbook()

    addWorksheet(wb2, "Dados")
    writeData(wb2, sheet = "Dados", inc_c, startRow = 1, startCol = 1)

    style_table(wb2, "Dados", inc_c, startRow = 1)
    colorize_rows_by_tipoerro(wb2, "Dados", inc_c, startRow = 1)
    apply_dropdown_por_linha(wb2, "Dados", inc_c, startRow = 1)

    pageSetup(wb2, sheet = "Dados", fitToWidth = 1, fitToHeight = FALSE)

    write_title_block(
      wb2, "Resumo",
      title = paste("RELATÓRIO DE QUALIDADE -", cnome),
      sub1  = paste0("Processado em: ", format(ref_hoje, "%d/%m/%Y")),
      sub2  = comparativo_centro,
      sub3  = taxa_txt,
      total_cols = max(12, ncol(inc_c))
    )

    pageSetup(wb2, sheet = "Resumo", fitToWidth = FALSE, fitToHeight = FALSE)
    saveWorkbook(wb2, arq_centro, overwrite = TRUE)
  }
}
```

```
message("Reports by center generated")
```
